# Distinguishing active pediatric COVID-19 pneumonia from MIS-C

**DOI:** 10.1186/s12969-021-00508-2

**Published:** 2021-02-24

**Authors:** Daniel D. Reiff, Melissa L. Mannion, Nichole Samuy, Paul Scalici, Randy Q. Cron

**Affiliations:** 1 Division of Rheumatology, Department of Pediatrics, University of Alabama at Birmingham, 1600 7th Ave. S., CPPN #G10, Birmingham, AL 35233-1711 USA; 2 Division of Hospital Medicine, Department of Pediatrics, University of Alabama at Birmingham, 1600 7th Ave S, McWane Building, Suite 108, Birmingham, AL 35233 USA

## Abstract

**Importance:**

Active pediatric COVID-19 pneumonia and MIS-C are two disease processes requiring rapid diagnosis and different treatment protocols.

**Objective:**

To distinguish active pediatric COVID-19 pneumonia and MIS-C using presenting signs and symptoms, patient characteristics, and laboratory values.

**Design:**

Patients diagnosed and hospitalized with active COVID-19 pneumonia or MIS-C at Children’s of Alabama Hospital in Birmingham, AL from April 1 through September 1, 2020 were identified retrospectively. Active COVID-19 and MIS-C cases were defined using diagnostic codes and verified for accuracy using current US Centers for Disease Control case definitions. All clinical notes were reviewed for documentation of COVID-19 pneumonia or MIS-C, and clinical notes and electronic medical records were reviewed for patient demographics, presenting signs and symptoms, prior exposure to or testing for the SARS-CoV-2 virus, laboratory data, imaging, treatment modalities and response to treatment.

**Findings:**

111 patients were identified, with 74 classified as mild COVID-19, 8 patients as moderate COVID-19, 8 patients as severe COVID-19, 10 as mild MIS-C and 11 as severe MIS-C. All groups had a male predominance, with Black and Hispanic patients overrepresented as compared to the demographics of Alabama. Most MIS-C patients were healthy at baseline, with most COVID-19 patients having at least one underlying illness. Fever, rash, conjunctivitis, and gastrointestinal symptoms were predominant in the MIS-C population whereas COVID-19 patients presented with predominantly respiratory symptoms. The two groups were similar in duration of symptomatic prodrome and exposure history to the SARS-CoV-2 virus, but MIS-C patients had a longer duration between presentation and exposure history. COVID-19 patients were more likely to have a positive SAR-CoV-2 PCR and to require respiratory support on admission. MIS-C patients had lower sodium levels, higher levels of C-reactive protein, erythrocyte sedimentation rate, d-dimer and procalcitonin. COVID-19 patients had higher lactate dehydrogenase levels on admission. MIS-C patients had coronary artery changes on echocardiography more often than COVID-19 patients.

**Conclusions and relevance:**

This study is one of the first to directly compare COVID-19 and MIS-C in the pediatric population. The significant differences found between symptoms at presentation, demographics, and laboratory findings will aide health-care providers in distinguishing the two disease entities.

## Introduction

The pandemic of severe acute respiratory syndrome coronavirus 2 (SARS-CoV-2), causing coronavirus disease 2019 (COVID-19), has swept over the world with global estimates of 39 million cases and over 1.1 million deaths. As of writing, the US has seen over 6.5 million cases of COVID-19 with greater than 200,000 deaths. Children account for over 500,000 of the overall case burden, but thankfully less than 0.1% of deaths [1]. COVID-19 in the pediatric population has been shown to produce milder symptoms and a less severe disease course than in the adult population, with predominant symptoms of cough, fever, and pharyngitis [2] [3]. However, in addition to acute COVID-19, a clinically distinct constellation of symptoms has been seen in children as a sequela of past SARS-CoV-2 infection. Multisystem inflammatory syndrome in children (MIS-C) is a disease process notable for hyper-inflammation, fever, and Kawasaki-like symptoms, with severe cases progressing to cardiovascular shock [4].

In Alabama, COVID-19 cases have reached greater than 148,000 statewide with over 2500 deaths [5]. As compared to other areas, where experience with this novel disease saw a peak of COVID-19 cases followed later by a peak in MIS-C cases, Alabama has seen a consistent and prolonged spread of COVID-19 and MIS-C cases occurring simultaneously [6]. This simultaneous spread has caused diagnostic uncertainty in the pediatric population, as each disease process can have non-specific signs, symptoms, and laboratory findings at presentation. The aim of this study is to present the COVID-19 and MIS-C experience at our institution, compare the two disease processes, and to help front line providers distinguish COVID-19 and MIS-C cases quickly and efficiently.

## Methods

### Patients

A retrospective chart review was completed on all patients, between the ages of 0 and 22 years, diagnosed and hospitalized with COVID-19 infection and MIS-C at Children’s of Alabama Hospital in Birmingham, AL from April 1 through September 1, 2020. Patients were identified from the electronic medical record using International Classification of Diseases, tenth edition (ICD-10) codes for active COVID-19 pneumonia and MIS-C (ICD-10 U07.1), and further verified using positive virologic and serologic data.

### Clinical and laboratory evaluation

All clinical notes were reviewed for documentation of COVID-19 pneumonia or MIS-C and positive SARS-CoV-2 polymerase chain reaction (PCR) or IgG antibodies (Abbot Architech SARS-CoV-2 IgG test to the nucleocapsid protein). Clinical notes and electronic medical records were reviewed for patient demographics, presenting signs and symptoms, prior exposure to or testing for the SARS-CoV-2 virus, laboratory data, imaging, treatment modalities and response to treatment.

### Case definition and classification

Active COVID-19 pneumonia and MIS-C cases were verified for accuracy using current US Centers for Disease Control case definitions. From this point, active COVID-19 pneumonia will be simply referred to as COVID-19. COVID-19 cases were classified as mild, moderate, or severe. Mild cases were defined as asymptomatic or had fever as the only presenting symptom. Moderate cases were defined as patients requiring simple or high flow nasal cannula (up to 2 ml/kg of liter flow and 100% oxygen concentration). Severe cases required positive pressure ventilation and/or vasopressor support. MIS-C cases were classified as mild or severe, with mild MIS-C cases defined as requiring no respiratory support or low−/high-flow nasal cannula. Severe MIS-C cases were defined as requiring positive pressure ventilation and/or vasopressor support.

### Statistical analysis

Continuous variables are reported as medians and interquartile ranges. Categorical variables are summarized as frequencies and percentages. Statistical comparisons between the total active COVID-19 pneumonia and MIS-C populations and the severe COVID-19 and severe MIS-C populations were determined at a *p*-value of 0.05 using Fisher Exact and Kruskal-Wallis testing. Statistics were computed for all demographic, clinical, and laboratory features using Microsoft Excel. This study was approved by the University of Alabama at Birmingham institutional review board on September 8th, 2020 (decision number IRB-140306007).

## Results

### Study population and disease classification

**Fig. 1:**
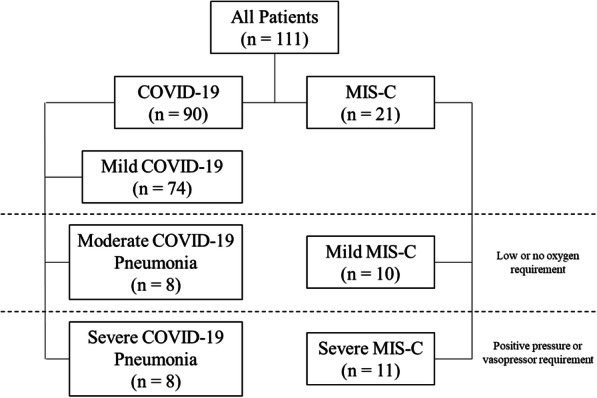
Study Population

111 patients were identified with diagnoses of COVID-19, MIS-C, or were found to be positive for SARS-CoV-2 PCR or IgG antibodies. 74 were classified as mild COVID-19 infections. 16 total patients were found to have active COVID-19 pneumonia, with 8 patients classified as moderate COVID-19 and 8 as severe COVID-19. An additional 21 patients during this period were hospitalized with MIS-C, with 10 classified as mild and 11 classified as severe (Fig. 1).

### Patient demographics and characteristics

Patients presenting with COVID-19 were a median age of 15.5 years (interquartile range (IQR) 8.8–17.3) and patients with MIS-C were a median age of 12 years (IQR 8–13) (Table 1). Most patients were male. Non-Hispanic Black patients made up the majority of all patients and the majority of the COVID-19 and MIS-C groups. Only 1 (6%) patient in the COVID-19 population was found to be previously healthy, where 19 (90%) MIS-C patients were healthy prior to illness onset and this difference persisted when limiting to severe COVID-19 and severe MIS-C patients. This difference in pre-existing conditions was noted to be significant. The COVID-19 patients had obesity, asthma, chronic lung disease, cancer, autoimmune disease, diabetes, congenital heart disease, and neurodevelopmental disorders as underlying diagnoses.

**Table 1:** Patient demographics and characteristics

	Active COVID-19 Pneumonia (*N* = 16)	Moderate COVID-19 Pneumonia (*N* = 8)	Severe COVID-19 Pneumonia (N = 8)	MIS-C (*N* = 21)	Mild MIS-C (*N* = 10)	Severe MIS-C (*N* = 11)	Total vs Total	Severe vs Severe
Age - years (IQR								
Median	15.5 (8.75–17.25)	15.5 (12.75–16.25)	13 (2.25–18.25)	12 (8–13)	10 (8–12.75)	12 (11–13)	0.1010	0.7726
Gender - no. (%)								
Male	11 (69)	6 (75)	5 (62.5)	12 (57)	4 (40)	8 (73)	0.5154	1.0000
Race - no. (%)								
White	4 (25)	0 (0)	4 (50)	6 (29)	3 (30)	3 (27)	–	–
Black	9 (56)	6 (75)	3 (37.5)	11 (52)	5 (50)	6 (55)	–	–
Other	3 (19)	2 (25)	1 (12.5)	4 (19)	2 (20)	2 (18)	–	–
Ethnicity - no. (%)								
Hispanic	3 (19)	2 (25)	1 (12.5)	4 (19)	2 (20)	2 (18)	–	–
Non-Hispanic	13 (81)	6 (75)	7 (87.5)	17 (81)	8 (80)	9 (82)	–	–
Previously Healthy - no. (%)								
Yes	1 (6)	0 (0)	1 (12.5)	19 (90)	9 (90)	10 (91)	< 0.00001	0.0012
Underlying Conditions - no. (%)								
Any	15 (94)	8 (100)	7 (87.5)	2 (10)	1 (10)	1 (9)	–	–
Obesity	6 (37.5)	5 (62.5)	1 (12.5)	0 (0)	0 (0)	0 (0)	–	–
Asthma	4 (25)	3 (37.5)	1 (12.5)	2 (10)	1 (10)	1 (9)	–	–
Chronic Lung Disease	1 (6)	0 (0)	1 (12.5)	0 (0)	0 (0)	0 (0)	–	–
Cancer	2 (12.5)	2 (25)	0 (0)	0 (0)	0 (0)	0 (0)	–	–
Autoimmune Disease	1 (6)	0 (0)	1 (12.5)				–	–
Diabetes	2 (12.5)	1 (12.5)	1 (12.5)	0 (0)	0 (0)	0 (0)	–	–
Congenital Heart Disease	2 (12.5)	0 (0)	2 (25)	0 (0)	0 (0)	0 (0)	–	–
Neurodevelopmental Dx	5 (31)	0 (0)	5 (62.5)	0 (0)	0 (0)	0 (0)	–	–

### Presentation and SARS-CoV-2 testing

Fever was a common, but statistically distinct presenting symptom for both COVID-19 patients and MIS-C patients (Table 2). However, there was no statistical difference between severe COVID-19 patients and severe MIS-C patients with respect to fever. The total COVID-19 population and severe COVID-19 patients were more likely to present with respiratory symptoms as compared to the total MIS-C population and severe MIS-C patients. The total and severe MIS-C patients were significantly more likely to present with gastrointestinal symptoms than total and severe COVID-19 patients. All 21 MIS-C patients had gastrointestinal symptoms on admission, with the most common being nausea, vomiting, diarrhea, and abdominal pain. Kawasaki disease (KD)-like symptoms of rash and conjunctivitis were seen more in the MIS-C groups, but no statistical difference was seen between MIS-C and COVID-19 patients when it came to mucosal changes (Table 2).

**Table 2:** Presentation and SARS-CoV-2 testing

	Active COVID-19 Pneumonia (N = 16)	Moderate COVID-19 Pneumonia (N = 8)	Severe COVID-19 Pneumonia (N = 8)	MIS-C (N = 21)	Mild MIS-C (N = 10)	Severe MIS-C (N = 11)	Total vs Total	Severe vs Severe
Symptoms on Presentation - no. (%)								
Fever	12 (75)	7 (87.5)	5 (62.5)	21 (100)	10 (100)	11 (100)	0.0276	0.0578
Respiratory Symptoms	14 (87.5)	6 (75)	8 (100)	1 (5)	1 (10)	0 (0)	< 0.00001	< 0.00001
Hypoxia	7 (44)	1 (12.5)	6 (75)	0 (0)	0 (0)	0 (0)	0.0011	0.0010
Cough	7 (44)	5 (62.5)	2 (25)	0 (0)	0 (0)	0 (0)	0.0011	0.1637
Shortness of Breath	9 (56)	4 (50)	5 (62.5)	1 (5)	1 (10)	0 (0)	0.0001	0.0048
Gastrointestinal Symptoms	6 (37.5)	3 (37.5)	3 (37.5)	21 (100)	10 (100)	11 (100)	< 0.00001	0.0048
Nausea/Vomiting	5 (31)	2 (25)	3 (37.5)	15 (71)	8 (80)	7 (64)	0.0220	0.3698
Diarrhea	2 (12.5)	2 (25)	0 (0)	11 (52)	5 (50)	6 (55)	0.0165	0.0181
Abdominal Pain	1 (6)	1 (12.5)	0 (0)	12 (57)	6 (60)	6 (55)	0.0016	0.0181
Rash	0 (0)	0 (0)	0 (0)	13 (62)	7 (70)	6 (55)	0.0001	0.0181
Conjunctivitis	0 (0)	0 (0)	0 (0)	14 (67)	8 (80)	6 (55)	< 0.00001	0.0181
Mucosal Changes	0 (0)	0 (0)	0 (0)	4 (19)	2 (20)	2 (18)	0.1182	0.4854
Length of symptoms prior to presentation - days (IQR)								
Median duration of symptoms prior to presentation	3 (1.75–6.25)	3.5 (2.75–7)	2 (1–3.75)	4 (3–5)	5 (3.25–5)	4 (3–4.5)	0.2902	0.2155
COVID-19 exposures								
Known COVID-19 exposure - no. (%)	8 (50)	5 (62.5)	3 (37.5)	6 (29)	4 (40)	2 (18)	0.3051	0.6027
Prior documented COVID-19 infection - no. (%)	N/A	N/A	N/A	4 (19)	2 (20)	2 (18)	–	–
Time between exposure/infection and admission - days (IQR)	6 (4.25–7.75)	7.5 (6.5–8)	3.5 (3.25–3.75)	29.5 (24.5–32)	28 (14–31)	31 (29.5–33)	0.0201	–
Level of Admission - no. (%)								
Intensive Care	13 (81)	5 (62.5)	8 (100)	12 (57)	1 (10)	11 (100)	–	–
Acute Care	3 (19)	3 (37.5)	0 (0)	9 (43)	9 (90)	0 (0)	–	–
Length of Stay – days (IQR)	14 (6.75–28.25)	9.5 (5.5–12.25)	29.5 (21.75–47)	5 (3–8)	3.5 (3.25–5)	7 (5.5–10)	0.0008	0.0023
SARS-CoV-2 Positivity - no. (%)								
PCR	16 (100)	8 (100)	8 (100)	8 (38)	5 (50)	3 (27)	0.0001	0.0010
IgG Antibodies - no./total no. (%)	0/0 (0)	0/0 (0)	0/0 (0)	16/17 (94)	8/9 (89)	8/8 (100)	–	–
Additional Infectious Agent - no. (%)								
Viral	1 (6)	1 (12.5)	0 (0)	0 (0)	0 (0)	0 (0)	0.4324	1.0000
Bacterial	3 (19)	1 (12.5)	2 (25)	1 (5)	1 (10)	0 (0)	0.2962	0.1637
Vasopressor Requirement - no. (%)								
Yes	5 (31)	0 (0)	5 (62.5)	11 (52)	0 (0)	11 (100)	0.3161	0.0578
No	11 (69)	8 (100)	3 (37.5)	10 (48)	10 (100)	0 (0)	–	–
Respiratory Support - no. (%)								
None	0 (0)	0 (0)	0 (0)	8 (38)	7 (70)	1 (9)	0.0056	1.0000
Low Flow Oxygen	5 (31)	5 (62.5)	0 (0)	5 (24)	2 (20)	3 (27)	0.7165	0.2281
High Flow Oxygen	3 (19)	3 (37.5)	0 (0)	2 (10)	1 (10)	1 (9)	0.6339	1.0000
Positive pressure ventilation	8 (50)	0 (0)	8 (100)	6 (29)	0 (0)	6 (55)	0.3051	0.0445

No difference was seen in length of symptoms prior to presentation. Eight (50%) total COVID-19 patients and 3 (37.5%) severe COVID-19 patients reported a known SARS-CoV-2 exposure preceding development of symptoms. Only 6 (29%) total and 2 (18%) severe MIS-C patients recalled a history of exposure, with 4 (19%) and 2 (18%) patients having documented prior COVID-19 infections, respectively. In COVID-19 patients with known exposures, the median time to symptom onset from the time of exposure was 6 days (IQR 4.3–7.8) and among the MIS-C patients the median time to symptom onset was 29.5 days (IQR 24.5–32). Only 8 (38%) total MIS-C patients were PCR positive. Of the 17 MIS-C patients tested for SARS-CoV-2 IgG, 16 (94%) patients were positive. More COVID-19 patients required positive pressure ventilation and more MIS-C patients were stable without oxygen therapy during hospitalization (Table 2). MIS-C patients improved faster than COVID-19 patients, with a shorter length of hospitalization for MIS-C patients.

### Laboratory findings

Creatinine levels on admission were similar between the groups, suggestive of no significant difference in the number of patients with acute kidney injury at presentation, as defined by normal ranges for age and gender (Table 3). MIS-C patients were noted to have significantly lower sodium levels on admission than COVID-19 patients, and finding that consistent with macrophage activation syndrome (MAS), as hyponatremia has been previously reported in the disease as well [7]. Albumin, transaminase, brain natriuretic peptide, and troponin levels were similar in COVID-19 and MIS-C patients.

**Table 3:** Laboratory and echocardiography results

	Active COVID-19 Pneumonia (N = 16)	Moderate COVID-19 Pneumonia (N = 8)	Severe COVID-19 Pneumonia (N = 8)	MIS-C (N = 21)	Mild MIS-C (N = 10)	Severe MIS-C (N = 11)	Total vs Total	Severe vs Severe
Laboratory findings on admission - no. (%)								
Acute kidney injury	5 (31)	3 (37.5)	2 (25)	8 (38)	2 (20)	6 (55)	0.7386	0.3521
Elevated liver enzymes	12 (75)	7 (87.5)	5 (62.5)	13 (62)	5 (50)	8 (73)	0.4912	1.0000
Lymphopenia	9 (56)	4 (50)	5 (62.5)	14 (67)	6 (60)	8 (73)	0.7332	1.0000
Thrombocytopenia	3 (19)	0 (0)	3 (37.5)	5 (24)	1 (10)	4 (36)	1.0000	1.0000
Laboratory values on admission - median value (IQR)								
Sodium (mmoL/L)	139 (136.75–141.25)	137.5 (135.75–138.25)	141.5 (139.75–142.25)	133 (131.75–134.25)	134 (133–136.5)	132 (130.3–133)	0.0004	0.0059
Creatinine (mg/dL)	0.78 (0.52–1.14)	0.73 (0.56–0.99)	0.78 (0.38–1.39)	0.79 (0.59–0.89)	0.6 (0.57–0.76)	0.83 (0.77–1.24)	0.9390	0.5357
Albumin (g/dL)	3.7 (3.25–4.15)	4 (3.7–4.3)	3.3 (3.05–3.7)	3.4 (3–3.6)	3.6 (3.25–3.68)	3 (2.6–3.5)	0.0821	0.2898
Alanine transaminase (U/L)	37.4 (18.8–58.05)	55.05 (23.33–153.13)	28.55 (16.4–44.03)	42.4 (20.9–65.35)	34.3 (15.2–52.8)	52.2 (27.73–73.6)	0.9746	0.1309
Aspartate transaminase (U/L)	54.5 (33.75–115.25)	60.5 (48–126.25)	44.5 (27.25–70.25)	43 (25.75–56.25)	34 (24.25–46)	43.5 (36.25–56.75)	0.2791	0.6251
Brain znatriuretic peptide (pg/mL)	46.85 (27.25–111.375)	10 (10–31.7)	101.4 (39.85–1281.85)	642.5 (47.1–1129.15)	40.75 (10–101.6)	1004.95 (229.5–1778.5)	0.7353	0.6184
Troponin (ng/mL)	0.01 (0.01–0.02)	0.01 (0.01–0.02)	0.02 (0.01–0.05)	0.1 (0.02–1.4)	0.01 (0.01–0.07)	0.24 (0.03–2.17)	0.3194	0.2689
C-reactive protein (mg/dL)	4.6 (1.41–11.11)	3.62 (1.27–4.95)	9.2 (1.43–11.9)	15.93 (11.17–23.1)	14 (6.21–18.14)	17.73 (11.98–27.62)	0.0015	0.0057
Erythrocyte sedimentation rate (mm/hr)	25 (9–40)	29.5 (13–38.5)	19 (12.5–35)	48 (38–58)	49.5 (40.75–59.25)	41 (38–58)	0.0031	0.0097
Procalcitonin (ng/mL)	0.77 (0.37–2.65)	0.37 (0.18–3.28)	1.32 (0.77–2.61)	11.51 (4.35–24.41)	2.18 (1.90–2.45)	13.51 (8.41–31.96)	0.0004	0.0013
White blood cell count (10^3/uL)	9.95 (4.54–13.85)	4.3 (2.39–7.39)	11.44 (9.74–22.61)	10.58 (8.44–12.78)	10.82 (8.1–11.84)	9.79 (8.88–14.92)	0.4903	0.3218
Absolute lymphocyte count (10^3/uL)	1.15 (0.59–1.42)	1.15 (0.53–1.32)	1.19 (0.83–1.51)	0.84 (0.5–1.51)	1.07 (0.91–1.75)	0.5 (0.44–0.74)	0.5774	0.1200
Hemoglobin (g/dL)	12.1 (10.6–14.73)	12.6 (11.35–14.78)	11.7 (10.58–14.13)	11.5 (10.4–11.9)	11.55 (10.8–12.13)	11.3 (10.1–11.8)	0.1010	0.3020
Platelet count (10^3/uL)	201 (164.75–263.5)	226.5 (181.25–276)	180.5 (77.25–239.5)	190 (155–225)	215 (165.75–271.3)	180 (129.5–201)	0.6567	0.9671
International normalized ratio	1.1 (1.1–1.25)	1.13 (1.08–1.17)	1.1 (1.1–1.35)	1.24 (1.18–1.4)	1.15 (1.1–1.2)	1.4 (1.22–1.4)	0.0842	0.0877
Ferritin (ng/mL)	472.6 (230.6–1982)	472.6 (230.6–683.4)	710.5 (246.55–1998.25)	520.1 (282.5–789.1)	461.9 (201.25–761.9)	520.1 (370.7–1032.5)	0.7632	0.9342
Lactate dehydrogenase (U/L)	667 (462–818)	767 (609.5–4861.5)	647 (503.25–780.25)	244 (200–299)	271.5 (216–454.8)	225 (184.5–264)	0.0005	0.0049
D-dimer FEU (ug/mL)	1.31 (0.61–1.94)	1.18 (0.65–1.29)	1.93 (1.21–2.03)	2.51 (1.58–3.25)	1.71 (1.37–2.35)	3.15 (2.42–3.79)	0.0236	0.0416
Fibrinogen (mg/dL)	447 (312.5–594)	529 (513–610)	373 (260.25–500)	544 (467–632)	561.5 (424.75–633.3)	489 (468.5–629)	0.2114	0.0987
Echocardiography findings - no./total no. (%)								
Decreased systolic function or ejection fraction	1/7 (14)	0/2 (0)	1/5 (20)	5/21 (24)	1/10 (10)	4/11 (36)	1.0000	0.6000
Coronary artery dilation	0/7 (0)	0/2 (0)	0/5 (0)	7/21 (33)	2/10 (20)	5/11 (45)	0.1414	0.1186
Z-score - median value (IQR)	N/A	N/A	N/A	2.15 (2.12–2.92)	2.12 (2.11–2.12)	2.81 (2.15–3.03)	N/A	N/A
Heart block	0/7 (0)	0/2 (0)	0/5 (0)	1/21 (5)	0/10 (0)	1/11 (9)	1.0000	1.0000

Inflammatory markers on admission differed significantly between all groups. Median initial C-reactive protein (CRP) was 4.6 mg/dL (IQR 1.4–11.1) in total COVID-19 patients compared to 15.9 mg/dL (IQR 11.2–23.1) in total MIS-C patients. This significant difference was also seen between median values of 9.2 mg/dL (IQR 1.4–11.9) in severe COVID-19 patients and 17.7 mg/dL (IQR 12–27.6) in severe MIS-C patients. Erythrocyte sedimentation rates (ESR) on admission were also different between groups, with an initial median value of 25 mm/hr. (IQR 9–40) in total COVID-19 patients, compared to 48 mm/hr. (IQR 38–58) in total MIS-C patients, and 19 mm/hr. (IQR 12.5–35) in severe COVID-19 patients versus 41 mm/hr. (IQR 38–58) in severe MIS-C patients. Lower values in severe disease is consistent with coagulopathy often seen in MAS [8] [9]. There was a significant difference between median procalcitonin levels on admission between total COVID-19 (0.8 ng/mL (IQR 0.4–2.6)) and total MIS-C patients (11.5 ng/mL (IQR 4.3–24.4)) (*p* = 0.0004) and among the severe COVID-19 (1.3 ng/mL (IQR 0.8–2.6)) and severe MIS-C patients (13.5 ng/mL (IQR 8.4–32)) (*p* = 0.0013).

White blood cell counts, absolute lymphocyte counts, frequency of lymphopenia, hemoglobin levels, platelet counts, and frequency of thrombocytopenia were similar between all groups. Ferritin levels were not significantly different between COVID-19 and MIS-C patients. Lactate dehydrogenase (LDH) levels were higher in COVID-19 and severe COVID-19 patients, with median levels of 667 U/L (IQR 462–818) and 647 U/L (IQR 503–780) at admission, as compared to the median levels of 244 U/L (IQR 200–299) and 225 U/L (IQR 185–264) seen in the total and severe MIS-C patients. Conversely, D-dimer levels were higher in the total and severe MIS-C populations on admission, with median levels of 2.5 μg/mL (IQR 1.6–3.3) and 3.2 μg/mL (IQR 2.4–3.8), respectively. This differed in comparison to levels of 1.3 μg/mL (IQR 0.6–1.9) and 1.9 μg/mL (IQR 1.2–2.0) seen in total and severe COVID-19 patients (*p* = 0.02, *p* = 0.04).

### Cardiac findings

All 21 MIS-C patients received echocardiograms on admission, but only 7 COVID-19 patients had an echocardiogram. One severe COVID-19 patient had decreased systolic function and ejection fraction on echocardiography. 5 (24%) MIS-C patients had decreased systolic function and ejection fractions, with 4 (36%) of those being in the severe subgroup. No COVID-19 patients had coronary dilation or aneurysms. 7 (33%) of MIS-C patients had dilation of the coronary arteries, with 5 (45%) of them classified as severe MIS-C. The median z-scores of the coronary arteries in those with dilation were noted to be 2.15 (IQR 2.1–2.9) in the total MIS-C population and 2.8 (IQR 2.2–3.0) in the severe subgroup.

## Discussion

### Patient demographics and characteristics

This comparison of COVID-19 and MIS-C in the pediatric population demonstrated some important differences which may be helpful as the SARS-CoV-2 pandemic continues. In our study, patients demonstrated a wide range of ages at disease onset with a slight male predominance, which is similar to data published in other case series throughout 2020 [3]. Although the age of our patients did not differ significantly between the two diseases, the age of MIS-C patients is helpful for distinguishing this disease process from KD, as KD is known to typically affect children < 5 years of age. Black and Hispanic patients were seen in our cohort at a higher proportion than the general population of Alabama. In the most recent data available from the US Census Bureau, in the state of Alabama, White persons make up 69.1% of the population, with Black (26.8%) and Hispanic (4.6%) persons at much lower percentages. At 56% of COVID-19 patients and 52% of MIS-C patients, Black children were over-represented in our study, as were Hispanic children (19%). This racial disparity in COVID-19 infection and sequelae has been well documented throughout the pandemic, with other studies finding similar overrepresentation of minorities [6]. It has been postulated that this disparity is due, in part, to the fact that members of racial and ethnic minorities are more likely to live in densely populated areas, work in essential industries, and dwell in multi-generational living arrangements [10].

When it comes to distinguishing active COVID-19 from MIS-C, one of the most striking differences was the underlying medical condition of the affected patient. Our study demonstrated that the presence of an underlying medical condition was more commonly seen in the active COVID-19 pneumonia population as compared to the MIS-C population. Although there are few studies with data showing the extent of underlying health conditions within these two disease processes, the disparity observed in our population is more distinct, as others have shown previously healthy children at 60–70% in MIS-C populations and children with underlying conditions in 25–75% of COVID-19 patients [4] [6] [11] [12]. Interestingly, despite the large clinic volume seen by our rheumatology department, only one patient out of the 111 identified for this study had an underlying autoimmune condition. This patient was hospitalized for severe COVID-19 pneumonia complicating her systemic lupus erythematosus and lupus nephritis. Just prior to her hospitalization, she had been receiving treatment for a significant flare of symptoms, so was on a high dose of systemic steroids and had recently completed a round of rituximab, likely contributing to her severe disease.

### Presentation and SARS-CoV-2 testing

Another differentiating factor between active COVID-19 and MIS-C was symptoms of our patients on presentation. It has previously been shown that fever is a common presenting symptom of MIS-C, as seen in 100% of patients in two large cohorts [4] [6]. We have shown that fever is a statistically significant symptom that can help to distinguish the two disease entities, as a lower proportion of total COVID-19 patients presented with fever. The COVID-19 groups were shown to present with respiratory symptoms, such as hypoxia, cough, and shortness of breath, at a significantly higher frequency than MIS-C patients. The prevalence of respiratory symptoms in MIS-C patients has not been well reported in the literature, but our COVID-19 population manifested these symptoms at a similar frequency as other studies [13]. Additionally, presentation with gastrointestinal symptoms of nausea, vomiting, diarrhea, and abdominal pain was shown to be more prevalent in the MIS-C groups than in COVID-19 patients. Our MIS-C group had a high prevalence of gastrointestinal symptoms, similar to that seen in other cohorts at a rate of 70–80% [6]. However, other studies have shown higher prevalence of gastrointestinal symptoms in COVID-19 patients [13]. KD-like symptoms are a defining characteristic of MIS-C, so it is not surprising that our MIS-C patients had a higher proportion of rash and conjunctivitis than our COVID-19 groups.

In both our COVID-19 and MIS-C groups, only 40–50% of patients admitted a known SARS-CoV-2 exposure and only 19% of MIS-C patients had a previously documented COVID-19 infection. Studies have shown that up to 20–30% of COVID-19 infections may be asymptomatic [14]. This fact may be contributory to our findings, but the small number of patients with known exposures may also be influenced by poor public health infrastructure and testing capabilities in the state of Alabama [5]. There was a significant difference seen in the time from known exposure to hospital presentation between the COVID-19 and MIS-C patients, but the utility of this information is limited if patients have no exposure history. If quick and widespread SARS-CoV-2 PCR testing were available, this would be extremely helpful in differentiating MIS-C from active COVID-19, as patients with COVID-19 were more likely than those with MIS-C to have a positive PCR on admission. Previous studies have shown that 40–60% of MIS-C patients are positive on PCR testing [6]. Positive SARS-CoV-2 antibody testing is also helpful, as 94% of MIS-C patients tested positive. Antibody testing does historically take longer to result, so it is questionable how helpful this would be at distinguishing COVID-19 and MIS-C at presentation. The need for vasopressors was not significantly different between groups, however respiratory requirement did differ between COVID-19 and MIS-C groups. A higher proportion of severe COVID-19 patients required positive pressure ventilation and most MIS-C patients did not need oxygen or respiratory support on admission.

### Laboratory and echocardiography findings

A strength of this study is that it is one of the first of its kind to directly compare laboratory values between pediatric COVID-19 and MIS-C patients. COVID-19 is known to have a specific constellation of laboratory findings including decreased albumin, high CRP, high LDH, lymphopenia, and high ESR [15]. In MIS-C patients, notable laboratory findings include elevated ferritin, d-dimer, and fibrinogen levels, in addition to elevated markers of cardiac damage, thrombocytopenia, and lymphopenia [16]. In our study, significant differences were found in many laboratory values on admission. MIS-C patients were found to have lower sodium levels, higher measures of inflammation (CRP, ESR and procalcitonin), and higher d-dimer levels on admission. COVID-19 patients were noted to have higher LDH levels than MIS-C patients. It is understandable that MIS-C would cause elevated inflammatory markers and d-dimer levels, as it is thought to be a disease process driven by post-infectious hyper-inflammation and endothelial damage [17]. No significant differences were seen in the liver and renal involvement, hematologic testing, or markers of cardiac dysfunction. This is interesting, because although lymphopenia, thrombocytopenia, and markers of cardiac dysfunction can be used to diagnose COVID-19 and MIS-C, they are not sufficient to distinguish the two disease entities. However, these findings may be helpful in distinguishing MIS-C from KD, as leukopenia and high brain natriuretic peptide levels are not features of KD [18]. Echocardiography may be helpful to differentiate COVID-19 and MIS-C, as MIS-C patients were the only ones to have coronary artery dilation, but statistical significance was unable to be determined due to the number of patients in the study who underwent echocardiogram.

### Limitations

One limitation of this study is the small sample size and that it is limited to a single institution, so there is some concern about generalizability. However, we were able to find statistical significance in some important characteristics and these results are supported by similar findings in the literature. Additionally, given the retrospective nature of our study and the novelty of MIS-C, diagnostic workup, imaging, and laboratory testing varied greatly in the cohort, which may have limited our comparison of these disease processes.

## Conclusion

COVID-19 disease and MIS-C are difficult to diagnose with their non-specific signs and symptoms, especially in geographic regions with simultaneous presentations of both diseases. It is important to have diagnostic tools to rapidly distinguish the two, as they have different treatments and can be severe in their outcomes. Our study has shown that there are differentiating factors between the two disease entities, including chronic medical issues, primary respiratory or gastrointestinal symptoms, rash and conjunctivitis, and laboratory abnormalities on admission including sodium, CRP, ESR, procalcitonin, LDH, and d-dimer levels. Armed with these findings, providers now have additional tools to employ on the front lines of this pandemic to appropriately diagnosis and treat COVID-19 and MIS-C in the pediatric population.

## Data Availability

The datasets analyzed during the current study are available from the corresponding author on reasonable request.
